# Before compassion: sympathy, tact and the history of the ideal nurse

**DOI:** 10.1136/medhum-2019-011842

**Published:** 2020-07-30

**Authors:** Sarah Chaney

**Affiliations:** Centre for the History of the Emotions, Queen Mary University of London, London E1 4NS, UK

**Keywords:** medical humanities, nursing education, cultural history

## Abstract

The word ‘compassion’ is ubiquitous in modern healthcare. Yet few writers agree on what the term means, and what makes it an essential trait in nursing. In this article, I take a historical approach to the problem of understanding compassion. Although many modern writers have assumed that compassion is a universal and unchanging trait, my research reveals that the term is extremely new to healthcare, only becoming widely used in 2009. Of course, even if compassion is a new term in nursing, the concept could have previously existed under another name. I thus consider the emotional qualities associated with the ideal nurse during the interwar period in the UK. While compassion was not mentioned in nursing guidance in this era another term, ‘sympathy’, made frequent appearance. The interwar concept of sympathy, however, differs significantly from the modern one of compassion. Sympathy was not an isolated concept. In the interwar era, it was most often linked to the nurse’s tact or diplomacy. A closer investigation of this link highlights the emphasis laid on patient management in nursing in this period, and the way class differentials in emotion between nurse and patient were considered essential to the efficient running of hospitals. This model of sympathy is very different from the way the modern ‘compassion’ is associated with patient satisfaction or choice. Although contemporary healthcare policy assumes ‘compassion’ to be a timeless, personal characteristic rooted in the individual behaviours and choices of the nurse, this article concludes that compassionate nursing is a recent construct. Moreover, the performance of compassion relies on conditions and resources that often lie outside of the nurse’s personal control. Compassion in nursing—in theory and in practice—is inseparable from its specific contemporary contexts, just as sympathy was in the interwar period.

## Introduction

Compassionate care. Compassion fatigue. Compassion deficit. The word ‘compassion’ is everywhere in modern healthcare. In the UK, increased attention to the emotional side of care emerged most prominently during a public investigation into Mid-Staffordshire National Health Service (NHS) Foundation Trust (2010–2013). Local residents had been campaigning for an inquiry into Stafford Hospital since 2007, following a number of deaths after routine operations in appalling conditions. The subsequent investigation by Robert Francis QC outlined a number of causes of poor care including chronic staff shortages, bad management and low morale. An interim report, however, identified the Mid Staffs scandal as being ‘as much a story of very poor nursing care as of anything else; nursing care that lacked attentiveness and compassion’.1 While Francis was clear that this lack of compassion was related to other problems within the trust, the emphasis on uncaring nurses was picked up by policy-makers and the press alike; prime minister David Cameron even suggested that nurses should be ‘hired and promoted on the basis of having compassion as a vocation’.2

This notion of compassion as a natural aptitude or calling has permeated much contemporary healthcare literature. The final Francis Report (2013) recommended ‘an increased focus on a culture of compassion and caring in nurse recruitment, training and education’.3 This was adopted by NHS England who launched a 3-year strategy, ‘Compassion in Practice’, in early 2013, outlining six values to be upheld by nursing, midwifery and care staff. 4 Only compassion appeared in both the title of the report and the list of values in the framework, emphasising the centrality of this emotional capacity to modern healthcare. Some nursing authors, however, have argued that a contextual approach to the topic is important. While some of these still cite compassion as a key nursing value, it is presented not as the responsibility of the individual nurse, but as the product of a supportive environment.5

But what do we even mean by ‘compassion’? Compassionate care is presented in modern healthcare in an idealised and simplistic manner. It is defined as both emotion and action. Nursing authors tend to explain compassion as both an awareness of someone else’s distress and a desire to help relieve that distress.6 This individualised definition means that compassionate care is presented as something that simply happens, so long as the right people with the right qualities are in post. Yet even if one accepts this definition of compassion, there are many ways in which an urge to help others can be thwarted. As Smajdor pointed out in her review of the ethics of compassion, the Francis Report includes examples of many healthcare workers who were deeply distressed by conditions in Stafford Hospital, yet felt powerless to change them.7 If compassion does mean the capacity to be moved by suffering and wish to relieve it, then this example indicates why compassion in itself is not enough to produce good care. A focus on this individual trait can also mean that struggles by healthcare workers to manage challenging environments or a lack of material resources is perceived by others as a failure to care.8 Indeed, Flores and Brown suggest that healthcare scandals may have become more common ‘not in spite of, but because of’ growing expectations of compassionate care.9 Compassion in this context risks becoming ‘little more than a rhetorical and political device’ rather than a concept that can change or improve how services are delivered and experienced.’10

In this article, I take a historical approach to the problem of understanding compassion, rooted in the history of emotions. A relatively new field, the history of emotions emerged from the work of scholars like Reddy and Rosenwein in the early 2000s.11 The central tenet is to consider emotions themselves as historically and culturally constituted. Charting the changes in emotional landscapes through historical sources might include an investigation of shifts in the language used to describe feelings, different ways of representing, experiencing or understanding types of emotion or changing expectations about feelings in different cultures. Throughout history, countless scientists have tried and failed to pin emotion down to one specific thing: yet the physiological, psychological and experiential elements of emotion cannot be easily disentangled, as James found in his influential 1884 essay ‘What is an Emotion?’12 These efforts to understand and explain emotion are just as historically situated as the emotions themselves. In Norbert Elias’ influential sociological overview, *The Civilizing Process,* for example (first published in 1939), Elias took Victorian evolutionary models of progress and early 20th century psychological approaches to emotion as a simple reflection of reality. Human societies, under this model, progress from smaller to larger and more complex units, a process which is explained as civilisation: for Elias, ‘civilising emotions’ follow this same process to transform society.13 Yet this is as much a reflection of Victorian ideas of progress as an explanation of social change. As Boddice puts it in his recent overview of the field, ‘emotions are at once the effects of historical circumstances and a cause of their change’.14

Here, I follow a historical approach to look in detail at changes in feeling rules and expectations in nursing. Exploring a different period from our own effectively highlights the ways in which our modern expectations and understandings of emotion are neither obvious nor natural: they too are socially and culturally constituted. A historical approach to the topic sheds light on the consequences of our modern use—for better or worse—of these models of emotion. If, as many modern clinical writers have assumed, compassion really is an essential individual trait found in carers, one would expect to find reference to it throughout nursing history. On the contrary, the term is extremely new to healthcare, even though the word itself is not a new one. Compassion began to appear in association with nursing in the late 1980s, and only became a widely used term in 2009.

In this article, I look at the very different feeling rules in place in an earlier period. I focus primarily on the interwar period, with context from previous decades where necessary: early 20th century nurse leaders remained heavily influenced by Victorian reformers like Florence Nightingale and Ethel Gordon Fenwick, even if they did not have precisely the same expectations of nurses that Victorian commentators had. While historians have paid a great deal of attention to the role of Victorian nursing reformers in shaping a vocational view of nursing, embedded in so-called ‘natural’ female traits, rather less attention has been paid to models of nursing in the early twentieth century. The interwar period, in particular, is often neglected in histories of nursing, with the Nurses’ Registration Act of 1919 characterised either as the culmination of the work of nursing reformers or having little practical impact on a timeless nursing craft.15 This may be in part because nursing remained strongly associated with individual qualities, based on a class ideology of ‘moral character’.16 Yet through the foundation of a General Nursing Council (GNC) and the introduction of a universal exam for nursing, the Registration Act directed increased attention to defining what nursing was, as well as bringing into the foreground a pre-existing conflict between nursing as a scientific profession and as a female vocation.17 This period also saw a greatly increased number of textbooks and journals written by nurses for nurses, in comparison to the late 19th century, when nursing textbooks were often written by doctors.18 The achievement of universal examination and registration of nurses (even if training itself was not yet standardised) sparked increased interest in defining the ‘good nurse’, a definition also shaped by the changing place of women and the experiences of the First World War.

First, then, I look at how and why compassion became linked to healthcare, arguing that efforts to differentiate nursing from medicine through a patient-centred concept of care from the 1980s on led to a renewed focus on the individual qualities of the carer. Of course, even if compassion is a new term in nursing, the emotion it describes could have previously existed under another name. The Victorian and Edwardian periods saw several new terms brought into being relating to fellow feeling: altruism in 1851 as a secular form of sympathy and empathy in around 1909.19 I go on to consider the emotional qualities associated with the ideal nurse during the inter-war period in the UK. While compassion was not mentioned in nursing guidance in this era, ‘sympathy’ made frequent appearance. The interwar concept of sympathy, however, differed significantly from the modern one of compassion. Sympathy was focused around patient management, while the modern emphasis on compassion is usually on patient satisfaction or choice. Sympathy was not an isolated concept, and was usually linked to other traits. In the interwar era, it was most often connected with the nurse’s tact or diplomacy. A closer investigation of this link reinforces the emphasis laid on patient management in this period, as well as the way class differentials in emotion between nurse and patient were emphasised in the efficient running of hospitals.

I present these two periods (the interwar period and the modern day) as a comparison, not as a chronology of development. I am not trying to describe or explain a shift from sympathy to compassion in nursing but recognising that the two are quite separately constituted models of care. Although contemporary healthcare policy assumes ‘compassion’ to be a timeless, personal characteristic, rooted in the individual behaviours and choices of the nurse, this article concludes that compassionate nursing is a recent construct. Moreover, the performance of compassion relies on conditions and resources that lie outside of the nurse’s personal control. Just as sympathy and tact in the interwar era shed light on models of hospital managements and expectations of gendered care, so too does compassion need to be explored in relation to the context of modern healthcare, and not simply as an expectation laid on individual nurses.

## Becoming compassionate: a new model for healthcare in the 21st century

Before the 21st century, ‘compassion’ was rarely used to describe healthcare in the UK. In nursing textbooks and journals from the 1880s to the Second World War, the term is significant only in its absence. Even in more recent years, the word compassion has been at most peripheral to healthcare. The British Nursing Index contains just six references to compassion for the whole of the 1980s, the first 10 years of the database’s coverage. In the most recent decade (2010–2019) this leapt to 1645, well out of proportion to the overall increase in articles.20 A sharp spike in use occurred in 2013, the year ‘Compassion in Practice’ was launched and the final Francis Report published. However, a gradual increase had taken place before this. This change also occurred outside the UK, with growing use of the term compassion in international databases such as the Cumulative Index of Nursing and Allied Health Literature.21 This suggests that the UK story is part of a more general shift in western understandings of nursing care and changing attitudes to and ideas in nursing education and practice.

In a recent review article, Canadian researchers McCaffrey and McConnell explored this wider trend. Between 2000 and 2013, they found that there had been a particularly steep rise in attention to compassion in journal articles written in the UK. However, compassion had also simultaneously become a topic of international discourse: 9 of the 20 articles they reviewed were not by UK authors.22 What has contributed to this trend? Reflecting back on her seminal research into the emotional labour of nursing in 1984–1985, Smith suggested that an international emphasis on the emotional aspects of care emerged in the 1980s and 1990s, when relationships with patients became classed as ‘distinctly nursing work’.23 In 2008, the NHS Review, ‘High-Quality Care for All’, introduced compassion as an NHS value in the UK and proposed a ‘compassion index’ for nurses.24 Although the review stated that this value should apply to all NHS staff, it largely seems to have been picked up in nursing. A 2017 study by Bivins *et al* found that the phrase ‘compassionate care’ is today associated with nurses and not with doctors, surgeons or managers across professional journals and most UK newspapers.25

But just what are all these modern reviews and inquiries trying to measure? No one, it seems, can quite agree. Is compassion a form of emotional labour—a task-oriented or performative emotional state—or a virtue—a quality found in individual nurses?26 Jones and Pattison suggest that, like ‘community’, compassion functions as a ‘portmanteau concept … containing many desirable if undetermined meanings and being confined to very little that is specific’.27 Jones and Pattison go on to list a number of potential components of compassion in modern usage, which exist in unspecified amounts, including sympathy, pity, empathy, solidarity, feeling with or for others and putting oneself in someone else’s shoes. Historians of emotion have similarly shown the vague and expansive way compassion as a term has been used in other circumstances, especially politically. The only clear thing about compassion, as Berlant put it, is ‘that it implies a social relation between spectators and sufferers.’28 Indeed, this could be said to be both the most constant and most changeable aspect of compassion over the centuries. While the word compassion has been associated with a form of ‘fellow feeling’ since the 14th century, huge shifts in cultural and social norms across centuries and cultures mean that the way fellow feeling is interpreted, enacted and governed has altered countless times over the years. The terms in widespread use have also changed. In 18th century Europe, ‘sympathy’ was a marker of social difference: a means of communication among the elite that also functioned as a way of excluding outsiders.29 In the late Victorian era, meanwhile, the same term sympathy—and its new secular counterpart, ‘altruism’—became interpreted in evolutionary terms, as a sign of social progress.30 This meant that acts that were not previously regarded as compassionate on a personal level were reinterpreted as affective (and therefore civilised) practices. Boddice has shown, for example, how vivisection was reinterpreted by scientists as compassionate in the 1890s. Although the act required an insensitivity to the sufferings of an animal, the potential benefit to humanity was emphasised by these men as ‘true’ compassion.31

These changes in the way social emotions have been interpreted in different historical periods suggests that it would be a mistake to map modern ideas of compassion in healthcare onto historical nursing texts—or even onto earlier terms, such as sympathy or altruism. While it might be possible to chart and explain changes in language over time, this would need to be a much longer study of chronological change, well beyond the scope of this article. Terms, after all, alter in meaning as well as prominence and any study of changes in language would need to consider this wider picture.32 The contemporary understanding of compassion tends to assume that it is both feeling and action, although it has certainly not always been understood in that way. From the 14th to the 17th centuries, compassion generally described an emotion felt on behalf of someone else who suffers, with no reference to action at all. Even when it came to mean a shared feeling in the eighteenth century, this still did not imply accompanying support.33

Despite this, the idea that compassion is an unchanging trait is a surprisingly frequent claim in contemporary healthcare, even by those who are critical of the modern lack of definition. It is suggested that compassion ‘has a long legacy’ in nursing, usually dating back to Florence Nightingale.34 Yet the reality is more complicated than this connection suggests. In her research on the history of nursing education, for example, Bradshaw rightly recognises that nursing textbooks from the late 19th century to the 1960s laid great importance on the individual qualities of the nurse. Yet, by claiming that the character these texts outlined is the same as the modern notion of compassionate care, Bradshaw ignores the emphasis on gender and class in these texts. By viewing compassion as an unchanging, timeless entity, she also assumes that the long lists of traits that Victorian and Edwardian nurses were expected to develop in their training—‘cleanliness, neatness, obedience, sobriety, truthfulness, honesty, punctuality, trustworthiness, quickness and orderliness’—were intended to make probationers compassionate, although this was not a claim made at the time.35 In a more critical analysis of the modern focus on compassion, Traynor suggests the roots of this approach emerged from the restricted roles available to women in the nineteenth and early twentieth centuries. To differentiate nursing from male professions, matrons framed care as a vocational calling, based on a desire to serve. In the 20th century, as nursing became increasingly technical, nursing leaders attempted to distinguish nursing from medicine by claiming the nurse was the patient’s advocate.36 Despite situating his views in social context, Traynor’s view bears some similarity to Bradshaw’s in that he assumes that we are talking about the same trait across a long historical period. But can we really assume that earlier models of sympathy in nursing were the same as the patient advocacy of the 1980s or the compassion of today? I argue that this was not the case, by looking in greater depth at the emotions that were expected of nurses in the decades before the NHS came into being. How different were these from the modern notion of compassion?

## Sympathy and fortitude: the ideal nurse in the interwar period

It was not new to the interwar period for a link to be made between nursing care and an individual nurse’s character or feelings. Yet rather than thinking of nurses as exemplifying something called ‘compassion’ or ‘sympathy’, writers in earlier historical periods cited different qualities. In 1752, the regulations of Manchester Infirmary ruled that nurses must behave with ‘tenderness’ to patients.37 Victorian nursing reformers, such as Florence Nightingale and Ethel Gordon Fenwick, tended to focus on ‘good character’ as part of a class-based vocational framework for nursing.38 Fenwick, who aimed to transform nursing into an elite profession, emphasised middle-class ideals of femininity, like sympathy, gentleness, patience and tact.39 Nightingale, who expected that probationers would continue to come from the working and lower middle classes (although managed by Lady matrons), emphasised the traits expected of the respectable Victorian working-class woman: cleanliness, orderliness, industry, honesty and thrift.40 The views of reformers did not necessarily match those of their recruits. According to Dingwall *et al*, paid nurses tended to see their work in practical terms, even though the elite women who employed them viewed nursing in moral terms. By the late 19th century, however, nursing had become ‘dominated by the values of the ladies’.41 This was reinforced by a ‘two-tier system’ of nursing. Until at least the 1930s ‘gentlewomen’ could enter special probationer schemes, undertaking a shorter period of training for which they paid their own maintenance fees.42 These women, trained for leadership, were expected to shape the moral character of working class recruits to emulate middle class values.43 The attributes expected of the ideal candidate for nursing were often linked to this background, as outlined at one College of Nursing meeting in 1920. The ideal nurse should have ‘grace and dexterity through games and exercises’, ‘pleasant speech, as well as something to say’, be able to speak at least one foreign language, keep accounts and write a good letter: all things obtained through an elite education, and thus requiring recruits from the wealthier classes.44 This class-based understanding of emotion had a much wider reach beyond nursing, of course. Many writers of the interwar period and earlier began from the view that ‘affect-control and self-constraint are generally more highly developed’ in the upper classes, as Norbert Elias assumed in *The Civilizing Process.45*

What, then, was the ‘ideal nurse’ described by other nurses in this period? A GNC examination question in the 1920s and 1930s asked entrants what were the necessary qualifications for a good or ideal nurse.46 Model answers written by senior nurses focused on moral and character traits—from neatness and avoiding gossip to kindness and cheerfulness. Textbooks on nursing thus tended to begin by describing this ideal nurse, emphasising the traits deemed most important to nursing practice. I analysed this section in all the interwar UK textbooks, career guides and model examination answers listed in Alice Thompson’s 1968 Bibliography of Nursing Literature to get a picture of which emotional and character traits were seen as essential to nursing at this time. The most common six traits listed in both the 1920s and 1930s were those that described a nurse’s relationship with patients and their relatives—sympathy and tact—those that were characteristic of the nurse’s manner—calm and gentle—and those that focused on a nurse’s relationship with authority—obedience and loyalty. These six traits were found alongside others that appeared with slightly less frequency—kindness, cheerfulness, courtesy, patience and courage. In total, I found 41 different emotion-based character traits connected with nursing at this time, which indicates just how difficult the nursing character was to pin down. Many were mentioned just once or twice. Indeed, of the six most popular traits, only two can be found in at least half the texts—these were sympathy and tact, on which this article focuses.

Sympathy was one of a set of qualities that had been associated with Victorian middle-class women, and in the interwar period continued to be linked with the elite model of nursing outlined above. ‘Sympathy and service is the province of women,’ stated Joseph Johnson in *Earnest Women,* a late Victorian volume designed to inspire educated young women in their adult lives. According to Johnson, this arose because women were physically weaker than men. This meant that they were less often able to aid others through action; instead a woman gave ‘the sympathy of her heart’.47 Johnson used Florence Nightingale as an example to prove how natural these traits were in ‘earnest women’. Johnson’s use of the ministering angel stereotype—exemplifying the passive yet emotional role he identified for women—had little to do with Nightingale’s *actual* work, which was more about the management of resources than the direct provision of care.48 The stereotype instead relied on a glorification of the (presumed) domestic self-sacrifice of wives and mothers—the so-called ‘angel of the house’.49 During the 19th century, religious and charitable work had also come to be seen as a natural extension of the female role for middle and upper class women.50 A vocational view of nursing fitted neatly into this ideal.

The term ‘sympathy’ in the late nineteenth century was often framed in religious terms: this, after all, was why agnostic writers such as Auguste Comte and Herbert Spencer had sought to replace the sentiment with Comte’s new term ‘altruism’ in the 1850s.51 In nursing, however, religion remained prominent whichever of the two terms was used. In 1924, Ethel Gordon Fenwick set altruism within a religious framework, with a rare use of the term in a nursing context. She insisted that it was a nurse’s altruism that enabled her to treat the souls as well as the bodies of her patients. If she did not possess the proper vocational desires, the nurse would leave her patients ‘unsolaced and uncomforted’, even if she was able to treat them physically.52 This spirit of service, then, was still understood to be a religious imperative in the 1920s. When the College of Nursing commissioned three stained glass windows as ‘symbolical paintings of nursing’ for the lecture hall of their new building in 1925, they draw on the three Christian virtues advocated by St Paul: faith, hope and charity.53 The ‘nursing virtues’ became faith, love (a frequent substitution for charity in late Victorian religious texts)54 and fortitude.55

The addition of fortitude is an interesting one. Fortitude became associated with nursing following the First World War. Neither fortitude nor associated words like courage appear in lists of traits attributed to the Nightingale model of nursing.56 Both, however, made an appearance in models of nursing in the interwar period, although inconsistently. As Cochrane put it, the trained nurse required a combination of traits from other professions: ‘The courage of the soldier, the loyalty and faith of the clergy, the tact and diplomacy of the barrister, together with the skill, initiative and resource of the medical profession’.57 It was no accident that the nurse’s courage was associated with the soldier’s. During the First World War, 17 000 trained nurses had served in the armed forces, working close to the front lines for the first time in Casualty Clearing Stations, on ships and trains as well as at base hospitals.58 The role nurses played in war led directly to the founding of the College of Nursing in 1916 and supported the introduction of state registration in 1919. The word ‘fortitude’ was also found in another symbolic depiction of nursing from the 1920s: Sir George Frampton’s monument to Edith Cavell, unveiled in London’s St Martin’s Place on 17 March 1920. Executed for treason in 1915 by the German military after confessing to her involvement in an undercover resistance network, Cavell’s death provoked international uproar, but made her an enduring icon of nursing. Such was Cavell’s popularity that, in 1932, when Madame Tussaud’s asked visiting children which historical figure from the waxwork museum they wanted to be like when they grew up, Cavell took first place.59

According to Dixon, Edith Cavell was one of the first British women to be celebrated for showing a so-called ‘stiff upper lip’. This phrase was still new to the English language. It appeared as an Americanism in the 1870s, and only became widely used in a British context around the time of the Boer War (1899–1902), with a meaning that ‘gradually settled on one central quality, namely the ability to put on a display of bravery and to hide one’s true feelings in times of trial and suffering’.60 This is just what Cavell was celebrated for a little over a decade later: not for showing the right emotions, but for showing no emotion at all. Cavell’s bravery and stoicism were remarked on in a variety of sources, from the chaplain who attended her before her death to the British press. The monument took a similar line, celebrating Cavell’s fortitude, humanity, devotion and sacrifice (one word appears on each side of the memorial). Some of these traits appear to perpetuate the ministering angel stereotype invoked by late Victorian writers like Johnson: the passivity of sacrifice, for example. There is a stark difference, however, between Johnson’s depiction of women as reliant on emotion in the absence of action and the celebration of Cavell for behaviour that hid, as much as it revealed, emotion. As Dixon notes, this period was a time when ‘modern women struggled to escape from the age-old idea that theirs was the lachrymose sex—soft, caring, soppy, sobbing, hysterical and manipulative.’61 Cavell was emblematic of this shift.

This shift is also reflected in the complex way that contemporary nursing textbooks dealt with sympathy. Unlike the modern compassion—or the womanly sympathy of the Victorian angel of the house—sympathy in the interwar period was not always deemed a positive or useful trait. Five texts mentioning sympathy went on to warn against the dangers of sentimentality in nursing: the most commonly mentioned of all undesirable traits. ‘There is a vast difference between quiet, knowledgeable, sympathetic help and frothy sentimentality,’ warned Cochrane, Matron of the Charing Cross Hospital, in 1930. ‘The calm demeanour of the true nurse does not indicate hardness and insensibility to suffering, only that her training has taught her that emotional outbursts do as much harm during a crisis as anything of a more actively dangerous character’.62 Cochrane’s calm nurse resembled the stoic Cavell, who felt emotion but did not show it, with restraint newly emphasised as a marker of professionalism in nursing. The emotional young woman did not make the best nurse, agreed Smith, former matron of the Withington Hospital in Manchester and GNC examiner. Smith reported disapprovingly that she often encountered those who ‘approach nursing with too much sentiment and do not realise what a very practical side there is to it’.63

This distinction between sentiment and practical nursing was also used by interwar nurses to emphasise the importance of standardised healthcare education. While suggesting that there was a ‘germ of truth’ in the old saying that nurses are ‘born and not made’, Charlotte Moles clarified that this needed to be combined with adequate training for ‘correct nursing is not instinctive but has to be learnt’. She explained this with the example of a person collapsing: the untrained woman might rush to help the sufferer by picking him up, while the good nurse would know ‘it may be really dangerous to do so’ and not be led by her instinctual emotions.64 Similarly, sister-tutor Alice Jackson talked of reminding the ‘warm-hearted student’ that sympathy or love was not enough to be a good nurse and emotion needed to be ‘directed by intelligence’ or risk causing a patient unnecessary pain by poor manipulation of a fractured limb or massage technique.65

Indeed, in this era, the nurse with ‘too sentimental a sympathy’ was presented as just as problematic as the ‘callous-minded and selfish nurse’.66 The psychologist Beatrice Edgell addressed this with a rare mention of ‘empathy’ in her book *Ethical Problems: An Introduction to Ethics for Hospital Nurses and Social Workers* (1929). Edgell taught at Bedford College for Women, where several postregistration courses for nurses were set up by the College of Nursing in the 1920s and she would certainly have come into contact with the elite end of the nursing spectrum. Edgell cautioned that ‘sympathy must never become empathy’: perhaps a surprising distinction for a modern reader.67 Empathy was a new term that had entered psychology from aesthetics in the early twentieth century.68 In the 1920s, empathy meant a projection of one’s own feelings into an object or person, and not an ability to read and respond to someone else’s emotions as we might understand the term today. Projection is certainly how Edgell defined it. Her caution continued that:

There is a danger that understanding the situation in which another finds himself, we shall read into that situation not his feeling but our own. It is one thing for a nurse to know and be moved by the emotion of her patient, but it is another for her to experience an emotion herself and project that emotion into the situation. Emotionality in this sense is to be avoided.69

The nurse thus needed an awareness of her patient’s emotions (sympathy) but should not assume that the patient’s feelings would be the same as what she was feeling (empathy). Others referred to this ability to read someone else’s feelings as ‘imaginative sympathy’, a quality that would allow the nurse ‘to enter within the patient’s experience, to suffer with him to the extent of detecting the particular points of pressure of his suffering’. Again, there were strict limits and ‘that imagination needs to be controlled and disciplined, that sympathy unsentimental and heartening, if it is not to make the contact too painful a one for the nurse, and too emotional a one for the patient.’70

## ‘The tact of the nurses’: emotions and patient management in the interwar hospital

How did this ‘imaginative sympathy’ function in practice? And what was the purpose of ensuring it was ‘controlled’ and ‘unsentimental’? The answer to these questions revolved around patient management. The nurse was required not only to keep her own emotions in check, but also to manage the patient’s feelings. To explore this further, we need to look at the other traits that were linked to ‘sympathy’ in nursing during this period. One aspect of interwar sympathy that distinguishes it still further from compassion is the close relationship between sympathy and tact in nursing textbooks of the time. The two usually appeared in relation to each other, though it was tact that was more often emphasised in indices and section headings. In Charlotte Moles’ *Nursing as a Career* (1933), tact was one of the four general qualifications required of a woman going into nursing, along with observation, accuracy and truthfulness. Sympathy—which, unlike tact, did not appear in the index—was something a nurse needed ‘a good store of’ but was not in itself a criterion for judging a suitable candidate for the profession.71 For Esther Fisher in 1937, sympathy was a subcategory of tact: a ‘little judicious sympathy’ was required to aid the nurse in the skilled persuasion of her patients.72 Sympathy in this context was more closely aligned with changing a patient’s behaviour to facilitate cure than in understanding a person’s individual needs.

Tact in its modern sense of an awareness of social niceties was a newer term than sympathy. This meaning entered the English language only in the early 1800s. Tact quickly came to show ‘refinement and good breeding’ in etiquette guides of the Victorian era: like sympathy, it was thus curiously mired in class.73 It was also described as a female trait.74 Routledge’s *Manual of Etiquette* described the requirements of a Victorian dinner-party hostess as: ‘tact and good breeding, grace of bearing, and self-possession in no ordinary degree’.75 In the early 20th century, the nursing elite drew on this assumption about the natural social skills of women to emphasise the specific abilities women could bring to nursing: ‘Honour, commonsense, discretion and tact are generally acknowledged to be peculiar to women.’ An editorial in the *Nursing Mirror* boldly claimed. ‘Then nurses should endeavour to acquire a double portion of these qualities.’76 This approach was not specific to nursing, but also emerged in other professions dominated by women, such as health visiting and social science fieldwork.77 Professional expertise could only be claimed by women, it seemed, when it was associated with so-called natural female qualities. But why did matrons require their staff to be skilled socially? This was attached to the expectation that nurses would maintain good order on the wards. Just as a Lady superintendent was assumed to have a good effect on the morals of her nurses, so nurses were expected to influence *patients* for the better, in terms of both good behaviour and emotional outlook.78 As Cuff and Pugh’s *Practical Nursing* stressed, ‘the presence of a refined and courteous woman is sufficient under ordinary circumstances to maintain order in a ward’.79 This refined nurse should not have ‘too easy a standard or moral discipline for the patient’: her role was not just to cure the patient physically, but improve him or her morally and spiritually as well.80

Until the introduction of the NHS, hospitals in Britain were primarily used by the working and lower middle classes. Cuff and Pugh’s ‘refined and courteous’ woman implied a middle-class nursing sister, able to maintain order through the authority of her rank. This is, after all, another reason why Victorian matrons had stressed the similarities between hospital and home management.81 The educated woman managed her servants in the home and both staff and patients in a hospital. Sometimes class differentials might cause challenges. A Midlands doctor, known as Luke, wrote to his niece Barbara during her nursing training about how to manage the ‘perplexing’ and ‘crude’ but ‘quite lovable’ outpatient mother.82 However, despite urging Barbara to respect these patients, Luke counselled her to ensure she carried out the doctor’s orders, even when these patients did not like them. As Luke concluded, ‘a hospital’s good repute depends far more on the tact of the nurses than it does on the skill of the doctors.’83 A nurse needed to understand her patients so that she could tactfully manipulate them: maintaining order, ensuring the best in patient care (in spite of the ‘pleading or threatening’ of the patient, who knew no better) and making the very reputation of the hospital.

It was not only with patients that tact was useful. Gamarnikow has outlined the way in which middle and upper class matrons at the turn of the twentieth century described ‘tact’ as a fundamental skill in managing difficult doctors or avoiding inappropriate demands from their superiors.84 Gamarnikow also brings to attention an interesting aspect of the way nursing was framed in terms of emotional capacities; nurses themselves used assumptions about gendered traits—in this case tact—to draw professional boundaries. The importance placed on tact and discretion, however, also meant that nurses were often strict regulators of each other’s behaviour. It was not usually professional incompetence or even a lack of compassion or emotion that led to disciplinary action against a nurse in this period—the vast majority of cases discussed by the GNC Disciplinary Committee in the 1920s and 1930s related to sexual indiscretions, from nurses named in divorce cases to those with illegitimate children.85 The emphasis in these cases was one of trust: how could a nurse named in the divorce courts be trusted in a professional capacity, it was posited, when she had proven herself to be indiscrete? As I have shown elsewhere, however, attitudes in these cases did change as expectations on women shifted in the early twentieth century. The Nightingale nurse had faced instant dismissal for sexual indiscretion; however, this was no longer the case in the 1920s and 1930s.86 Although the GNC disciplinary committee continued to stress their desire to ‘purify the profession’, they nonetheless considered that there might be extenuating circumstances around love affairs, a single illegitimate child, or even an attempt to procure an illegal abortion.87 The existence of these disciplinary cases, however, does indicate that a nurse’s behaviour in her personal life continued to reflect on her professional character.

Unlike sympathy, tact was invariably seen as a positive trait in interwar nursing. It was a technique for managing the emotions of the patient, as well as those of the nurse. Pearce described tact as the ‘psychological factor’ in nursing. The ‘psychologically minded’ nurse was ‘willing to learn how physical suffering reacts on the mind, disturbing the emotions and the will in such a way that the patient is not normal while he is ill’.88 Sympathy thus also meant recognising that the patient might behave differently in illness. This emotional capacity inspired the nurse’s tactful behaviour, helping her to manage the patient efficiently. Tact, however, was also something that helped to keep sympathy within proper limits, associated as it was with the ‘calm and gentle’ manner of the professional nurse. As Cochrane put it, to be calm meant avoiding ‘emotional outbursts’.89 ‘Never get flurried or excited,’ warned Fisher, ‘as it conduces to lack of confidence on the part of the patient.’90 Douglas Hay Scott’s 4 vol *Modern Professional Nursing*, which was intended as a complete guide for students throughout their education, strongly emphasised nursing as a female profession, for care was ‘the peculiar gift of her sex’. Nonetheless, he also took the Cavell-inspired line, stressing that ‘in no circumstances should the nurse display her emotions to the patient’. Scott, who was not a nurse, went further than most matrons by concluding that this meant: ‘It is occasionally the good actress who succeeds best in restoring her patient to health.’91 While Scott was referring to the nurse’s ability to smile in ‘the midst of worry’, he nonetheless suggested that nurses might not actually need to feel sympathy, kindness or cheerfulness at all, but merely behave in a manner that conveyed these emotions to others. The desired result was the efficient management of the patient: tact and sympathy were simply skills that helped achieve this end.

## Conclusion

This increased emphasis on emotional restraint in interwar nursing textbooks was not necessarily echoed outside the nursing profession. Newspapers continued to present nurses in idealised, feminine terms, and nursing writers worried that this mismatch between expectation and reality caused some observers to reflect badly on nurses’ emotions. ‘Nurses are often said to be ‘hard’ and callous,’ wrote E. Maude Smith. ‘This is never true of a good nurse, but outsiders do not always realise that if a nurse did not control her feelings, she would be of little use to her patient’.92 Interwar nurses were caught in a bind, just like the modern girl who had ‘no time for tears’.93 As Dixon has shown, women in this period who expressed their emotions by weeping were regarded as hysterical and manipulative; those who did not were pitiless and uncaring. Smith and other matrons of her era tried to straddle this uncomfortable divide, making a distinction between the way a nurse’s behaviour might appear to others, and her own internal, yet invisible, feelings. While the trained nurse might maintain a stoic front, it was ‘never’ true that a good nurse did not feel anything. To be a professional nurse in this era was to restrain emotion, but to be a good and caring woman was to nonetheless feel the sympathy and devotion that had characterised the Victorian housewife.

As this article has shown, the model of emotions in nursing in the interwar period is not the same as the modern concept of compassion, indicating that compassion itself cannot be seen as an unchanging component of nursing. In the first place the word ‘compassion’ was not used to describe nursing in textbooks, journals, committee minutes or novels. Indeed, the link between nursing and compassion is a very modern one, emerging in the twenty-first century, most prominently in the UK. This UK interest in nursing emotion was, in part, due to a number of public healthcare scandals, of which the Francis Report and Mid Staffs Inquiry is one of the best known. Yet the wider context of this attention to compassion can be found in changes within healthcare systems (such as the move to a busier, target-driven model of care outlined by Pam Smith) and client-centred ideals, which focus on patient satisfaction as well as care outcomes.94 One of the most significant elements of this new interest in compassion, however, has been the assumption that it is not new. Compassion, we are told, is a universal and unchanging trait, which has always been important within nursing.

An exploration of nursing emotions in the interwar period, however, has shown that not only is compassion new but that even its seeming precursors, like sympathy, have changed over time as expectations of female behaviour altered. While the Victorian nurse’s sympathy was a passive womanly emotionality, the interwar nurse’s sympathy was characterised by restraint. The good nurse described by Maude Smith *had* sympathy but didn’t need to show it. She kept her emotions carefully within limits, something essential to her professional status: unlike other women, she was never sentimental. Tact—the nurse’s way of managing the behaviour of patients (and doctors)—was similarly twofold: appreciating and responding to the moods of others, and trying to channel those feelings to achieve a desired result. Like sympathy, tact was not something that others were expected to notice. This is quite different from the modern compassion which is strongly associated with patient satisfaction, deemed ‘central to how people perceive their care’.95 This sets compassion within a more modern focus on ‘person-centred’ care as a form of customer service in which quality of care is defined by the patient as much as the caregiver. In interwar hospitals, by contrast, efficiency and order were given considerably higher priority. Here, the emotional elements of care were thought to be best employed by nurses in managing the feelings of their patients, and ensuring these patients followed the doctor’s orders.

This distinction is an important one if we are to appreciate the way emotions are deployed in nursing today. Our modern view of the appropriate emotions in nursing is both consciously and unconsciously influenced by the history of attitudes to healthcare: the idea of nursing as a vocation, the gendering of care as a female pursuit and the regulation of the profession through attitudes to class. When we view the emotions in nursing as timeless and ignore the social context in which these feeling rules were created, we risk reinforcing the attitudes to gender and class embedded in these attitudes. This has wider implications for nursing practice: it can lead to a lack of attention to the emotional side of care in education and training, the devaluing of associated skills as ‘merely’ common sense or obvious and the absence of emotional support and mentoring for students and trained healthcare staff. Yet the understandings of emotional norms that new nursing recruits bring into the profession will vary depending on a range of demographic factors: their age, their cultural background, or the country in which they trained. Even within a group of nurses in the same cohort, views of compassionate practice will differ. Nursing research needs to address this lacuna in emotional models of care, which in the past has been perceived as either too difficult to define or not worth defining at all.96 After all, as this article has shown, the ways in which emotions are felt, understood and deployed in healthcare is not fixed or obvious. Exploring expectations about feeling in nursing in the interwar period has indicated that these say more about the prevailing culture and attitudes of that era than they do about individual nurses themselves. Today, the word compassion remains overused and little understood. If we are to continue to employ it at all, we need to be aware of the work the concept does historically, socially and politically, and avoid using it to draw assumptions about the moral character of the individual nurse.

## Patient and public involvement statement

It was not appropriate or possible to involve patients or the public in the design, or conduct, or reporting, or dissemination plans of this historical research.

## Data Availability

Data sharing not applicable as no datasets generated and/or analysed for this study. Bibliography provided with article. No further data produced.

## Notes

1. Inquiry, The Mid Staffordshire NHS Foundation Trust (2010), “Robert Francis (2013), Inquiry Report into Mid-Staffordshire NHS Foundation Trust,”, 417.

2. Denis Campbell, “David Cameron's prescription for NHS failings: target pay of nurses”, *The Guardian,* February 6, 2013, https://www.theguardian.com/society/2013/feb/06/david-cameron-nhs-nurses [visited 23/04/19] For examples of wider media condemnation, see Alistair Hewison and Yvonne Sawbridge, *Compassion in Nursing: Theory, Evidence and Practice* (London: Palgrave Macmillan, 2016), 3.

3. Inquiry, The Mid Staffordshire NHS Foundation Trust (2010), “The Mid Staffordshire NHS Foundation Trust Public Inquiry : Final Report. Presented to Parliament Pursuant to Section 26 of the Inquiries Act 2005” (London, 2013), 76.

4. Jane Cummings and Viv Bennett (2012), “Compassion in Practice: Nursing, Midwifery and Care Staff, Our Vision and Strategy,” 2012, https://www.england.nhs.uk/wp-content/uploads/2012/12/compassion-in-practice.pdf. The 6 Cs remained part of the follow-on framework, alongside the ‘10 commitments’, in 2018. Susan Aitkenhead, Liz Fenton, and “Leading Change (2018), “Leading Change, Adding Value: A Framework for Nursing, Midwifery and Care Staff,” 2018, 16, https://www.e-lfh.org.uk/programmes/leading-change-adding-value/.

5. Alistair Hewison and Yvonne Sawbridge (2016), *Compassion Nurs. Theory, Evid. Pract.*; Pam Smith, *The Emotional Labour of Nursing Revisited* (Basingstoke: Palgrave Macmillan, 2012); Anna Smajdor, “Reification and Compassion in Medicine: A Tale of Two Systems,” *Clinical Ethics* 8, no. 4 (December 18, 2013): 111–18, https://doi.org/10.1177/1477750913502620; Michael Traynor (2017), *Critical Resilience for Nurses* (Abingdon, Oxon: Routledge).

6. Graham McCaffrey and Shelagh McConnell (2015), “Compassion: A Critical Review of Peer-Reviewed Nursing Literature,” *Journal of Clinical Nursing* 24: 3008.

7. Anna Smajdor (2013), “Reification and Compassion in Medicine: A Tale of Two Systems,” 4.

8. Josiane Carine Tantchou (2018), “The Materiality of Care and Nurses’ ‘Attitude Problem,’” *Science, Technology & Human Values* 43, no. 2: 270–301.

9. Ruben Flores and Patrick Brown (2018), “The changing place of care and compassion within the English NHS: an Eliasean perspective”, *Social Theory and Health,* 16, 168.

10. Stephanie Tierney et al. (2017)., “Appraising the Situation: A Framework for Understanding Compassionate Care,” *Journal of Compassionate Health Care* 4, no. 1: 1.

11. William Reddy (2001), *The Navigation of Feeling: A Framework for the History of Emotions* (Cambridge: Cambridge University Press); Rosenwein, 2002, “Worrying About Emotions in History”, *The American Historical Review*, 107, no. 3, 821-45; Jan Plamper (2012), *The History of Emotions: An Introduction* (Oxford: Oxford University Press).

12. William James (1884), “What is an Emotion?” *Mind*, 9, no. 34, 188-205.

13. Norbert Elias (2000), *The Civilizing Process*, translated by Basil Blackwell, (Oxford: Blackwell), 436

14. Rob Boddice (2018), *The History of Emotions,* (Manchester: Manchester University Press), 18.

15. For the former see Rafferty (1993), “Decorous Didactics: Early Explorations in the Art and Science of Caring, circa 1860-90”; Robert Dingwall, Anne Marie Rafferty, and Charles Webster (1988), *An Introduction to the Social History of Nursing*. For the latter see Barbara Melosh (1982), *“The Physician’s Hand”: Work Culture and Conflict in American Nursing* (Philadelphia: Temple University Press); Olson, 1993, “Apprenticeship and Exploitation: An Analysis of the Work Pattern of Nurses in Training, 1897-1937,” *Social Science History* 17, no. 4: 559–76.

16. Also recognised in other histories of nursing, such as Robert Dingwall, Anne Marie Rafferty, and Charles Webster (1988), *An Introduction to the Social History of Nursing*, 82.

17. For more on this conflict see Rafferty (1993), “Decorous Didactics: Early Explorations in the Art and Science of Caring, circa 1860-90.”

18. Alice M.C Thompson (1968), *A Bibliography of Nursing Literature, 1859-1960, with an Historical Introduction* (London: Library Association for RCN), xvii.

19. Thomas Dixon (2008), *The Invention of Altruism: Making Moral Meanings in Victorian Britain,* (Oxford: Oxford University Press); Susan Lanzoni (2018), *Empathy: A History* (New Haven; London: Yale University Press)

20. “British Nursing Index (2019) British Nursing Index,” accessed December 16, 2019, https://www.proquest.com/products-services/bni.html.

21. CINAHL Complete (2019) “Full-Text Nursing Journals | EBSCO,” accessed December 16, 2019, https://www.ebscohost.com/nursing/products/cinahl-databases/cinahl-complete.

22. Graham McCaffrey and Shelagh McConnell (2015), “Compassion: A Critical Review of Peer-Reviewed Nursing Literature,” 3007.

23. Pam Smith (2012), *The Emotional Labour of Nursing Revisited*, 17.

24. Smith, 22.

25. Roberta Bivins, Stephanie Tierney, and Kate Seers (2017), “Compassionate Care: Not Easy, Not Free, Not Only Nurses,” *BMJ Quality & Safety*: 1–4.

26. For more on emotional labour, see Arlie Russell Hochschild (2012), *The Managed Heart: Commercialization of Human Feeling* (Berkeley; Los Angeles; London: University of California Press); Pam Smith (2012), *The Emotional Labour of Nursing Revisited*. For an explanation of compassion as a virtue see June Jones and Stephen Pattison (2016), “Compassion as a Philosophical and Theological Concept,” in *Compassion in Nursing: Theory, Evidence and Practice*, ed. Alistair Hewison and Yvonne Sawbridge (2016) (London: Palgrave Macmillan), 45–46.

27. Jones and Pattison, 43.

28. Lauren Berlant (2004), ed., *Compassion : The Culture and Politics of an Emotion* (New York: Routledge), 1.

29. Daniel M Gross (2006), *The Secret History of Emotion: From Aristotle’s Rhetoric to Modern Brain Science* (Chicago; London: University of Chicago Press, 2006), 171–72; Ute Frevert (2016), “Empathy in the Theater of Horror, or Civilizing the Human Heart,” in *Empathy and Its Limits*, ed. Aleida Assman and Ines Detmers (London: Palgrave Macmillan, 2016), 87–91.

30. Thomas Dixon (2005), “The Invention of Altruism: Auguste Comte’s Positive Polity and Respectable Unbelief in Victorian Britain,” in *Science and Beliefs: From Natural Philosophy to Natural Science, 1700 - 1900*, ed. David M Knight and Matthew Eddy (Aldershot; Ashgate), 195–211.

31. Boddice (2016), *The Science of Sympathy* (Urbana, Chicago and Springfield: University of Illinois Press), 72. See also Paul White (2011), “Darwin Wept: Science and the Sentimental Subject,” *Journal of Victorian Culture* 16, no. 2: 195–213.

32. For readers who are interested in this type of approach, I recommend Dixon, *The Invention of Altruism* and Lanzoni, *Empathy: A History.*

33. From the 14^th^ to the 17^th^ century, compassion generally described an emotion felt on behalf of someone else who suffers, with no reference to action at all. Pedwell, 6.

34. Those who have made the claim include writers who continue to emphasise an individualised notion of care, such as Ann Bradshaw (2011), as well as those arguing for the importance of recognising the contextual factors inherent in compassion, including Papadopoulous and Ali and Tierney et. al. Ann Bradshaw, “Compassion: What History Teaches Us,” *Nursing Times* 107, no. 19–20: 12; Stephanie Tierney et al. (2016) et al., “Measuring Compassionate Care: Views of Healthcare Staff,” *Nursing Management* 23, no. 8: 22; Irena Papadopoulos and Sheila Ali (2016), “Measuring Compassion in Nurses and Other Healthcare Professionals: An Integrative Review,” *Nurse Education in Practice* 16: 133.

35. Ann Bradshaw (2001), “Compassion: What History Teaches Us,” 12. See also Ann Bradshaw, *The Nurse Apprentice, 1860-1977* (Aldershot: Ashgate, 2001).

36. Traynor (2017), *Critical Resilience for Nurses*, 36–39.

37. Robert Dingwall, Anne Marie Rafferty, and Charles Webster (1988)., *An Introduction to the Social History of Nursing* (London: Routledge), 11. See also Anne Borsay and Billie Hunter (2012)., *Nursing and Midwifery in Britain since 1700* (Basingstoke: Palgrave Macmillan), 31–35.

38. Christine Hallett (2012), “Forging a Profession (1830 - 1920),” in *Nursing and Midwifery in Britain since 1700*, ed. Anne Borsay and Billie Hunter (2012) (Basingstoke: Palgrave Macmillan), 61–64.

39. Rafferty (1993), “Decorous Didactics: Early Explorations in the Art and Science of Caring, circa 1860-90,” in *Nursing: Art and Science1* (London: Chapman & Hall), 48–60.

40. On Nightingale, see Monica Baly (1997), *Florence Nightingale and the Nursing Legacy*, 2nd edition (London: Whurr), xii. For more on the class-specific ideologies about women’s place in June Purvis, *Hard Lessons: The Lives and Education of Working-Class Women in Nineteenth-Century England* (Cambridge: Polity Press, 1989), 59–70.

41. Robert Dingwall, Anne Marie Rafferty, and Charles Webster (1988), *An Introduction to the Social History of Nursing*, 44–47. See also Sue Hawkins (2010), *Nursing and Women’s Labour in the Nineteenth Century* (Abingdon, Oxon: Routledge), 31.

42. J Brooks (2001), “Structured by Class, Bound by Gender: Nursing and Special Probationer Schemes, 1860-1939,” *International History of Nursing Journal* 6, no. 2: 16–18.

43. Brooks, 17.

44. June Purvis (1989), *Hard Lessons: The Lives and Education of Working-Class Women in Nineteenth-Century England*, 60.

45. Norbert Elias (2000), *The Civilizing Process*, 396.

46. Felicie Norton (1936), *Preliminary Questions and Answers*, 4th edition (London: Faber and Faber), 228; Doris Tayler (1926), *General Nursing Questions and Answers*, 1st edition (London: Faber and Gwyer (The Scientific Press)), 20. This question had disappeared from at least one 1940 exam book.

47. Joseph Johnson (1898), *Earnest Women: Their Efforts, Struggles and Triumphs* (London: T. Nelson), 88. See also Anne Summers (1988), *Angels and Citizens: British Women as Military Nurses 1854-1914* (London: Routledge and K. Paul), 3.

48. Robert Dingwall, Anne Marie Rafferty, and Charles Webster (1988), *An Introduction to the Social History of Nursing*, 40; Baly, *Florence Nightingale and the Nursing Legacy*.

49. Coventry Patmore (1863), *The Angel in the House* (London and Cambridge: Macmillan and Co). On the female ideal of self-sacrifice see also Purvis (1995), ed., *Women’s History: Britain, 1850 - 1945* (London: UCL Press), 2.

50. Judith R Walkowitz (1992), *City of Dreadful Delight : Narratives of Sexual Danger in Late-Victorian London* (London: Virago), 52–58.

51. Altruism, like the modern compassion, was defined primarily by action, which made the emotional component similarly difficult to characterise by contemporaries. Was an altruistic act defined by its result, they wondered, or the feeling behind it (whatever the actual outcome)? Thomas Dixon (2008), *The Invention of Altruism: Making Moral Meanings in Victorian Britain* (Oxford; New York: Oxford University Press).

52. Ethel Gordon Fenwick (1924), “Editorial: Nurses Keep Your Souls Alive!,” *The British Journal of Nursing* 72, no. 1866: 1. See also Bradshaw, *The Nurse Apprentice, 1860-1977*, 74–76.

53. King James Bible, Corinthians 1:13.

54. June Jones and Stephen Pattison (2016), “Compassion as a Philosophical and Theological Concept,” 47.

55. Royal College of Nursing, *College of Nursing Opening Brochure* (London: Royal College of Nursing, 1925), 21.

56. Florence Nightingale (1914), *Florence Nightingale to Her Nurses: A Selection from Miss Nightingale’s Addresses to Probationers and Nurses at the Nightingale School*, ed. Rosalind Nash (London: Macmillan and Co); Douglas Hay Scott, *Modern Professional Nursing, Vol. 2* (London: Caxton, 1936), 185.

57. Mary S Cochrane (1930) *Nursing* (London: Geoffrey Bles), 9.

58. Christine Hallett (2014), *Veiled Warriors: Allied Nurses of the First World War* (Oxford: Oxford University Press).

59. Thomas Dixon (2015), *Weeping Britannia: Portrait of a Nation in Tears* (Oxford: Oxford University Press), 215.

60. Dixon, The Invention of Altruism: Auguste Comte’s Positive Polity and Respectable Unbelief in Victorian Britain, 202.

61. Dixon, *Weeping Britannia: Portrait of a Nation in Tears*, 216.

62. Cochrane, *Nursing*, 186.

63. E. Maude Smith (1929), *Notes on Practical Nursing* (London: Faber and Gwyer (The Scientific Press)), 9.

64. Charlotte L Moles (1933), *Nursing as a Career* (London: Pitman), 1.

65. Alice M Jackson and Katharine F. Armstrong (1934), *Teaching in Schools of Nursing* (London: Faber and Faber), 19.

66. Henry Pratt Newsholme (1934), “Nurse and Patient” (London: Guild of Health), 2.

67. Edgell (1929), *Ethical Problems: An Introduction to Ethics for Hospital Nurses and Social Workers* (London: Methuen), 134.

68. Susan Lanzoni (2018), *Empathy: A History* (New Haven; London: Yale University Press), 21–45.

69. Edgell (1929), *Ethical Problems: An Introduction to Ethics for Hospital Nurses and Social Workers*, 134.

70. Henry Pratt Newsholme (1934), “Nurse and Patient,” 8.

71. Charlotte L Moles (1933), *Nursing as a Career*, 4.

72. Esther Fisher (1937), *The Nurse’s Textbook: A Manual of Medical and Surgical Nursing* (London: Faber and Faber), 5.

73. *A Manual of Politeness, Comprising the Principles of Etiquette and Rules of Behaviour in Genteel Society for Persons of Both Sexes* (Philadelphia: W. Marshall & Co (1837)), 82.

74. Joan Burstyn (1984), *Victorian Education and the Ideal of Womanhood* (New Brunswick, N.J.: Rutgers University Press), 33–34.

75. George Routledge (1860), *Routledge’s Manual of Etiquette* (London; New York: Routledge), 26.

76. *Nursing Mirror*, 1909: 72 cited in Eva Gamarnikow (1991), “Nurse or Woman: Gender and Professionalism in Reformed Nursing 1860-1923,” in *Anthropology and Nursing*, ed. Pat Holden and Jenny Littlewood (Abingdon: Routledge), 127.

77. Daisy Payling (2019), ‘“The people who write to us are the people who don't like us:” Class, Gender and Citizenship in the Survey of Sickness, 1943-1952.’ *Journal of British Studies* (in press, 2019), http://repository.essex.ac.uk/24980/.

78. Robert Dingwall, Anne Marie Rafferty, and Charles Webster (1988), *An Introduction to the Social History of Nursing*, 64.

79. Herbert E Cuff and W. T. Gordon Pugh (1927), *Practical Nursing Including Hygiene and Dietetics* (Edinburgh: Blackwood), 4.

80. Newsholme, “Nurse and Patient,” 2.

81. Barbara Mortimer and Susan McGann (2011), eds., *New Directions in the History of Nursing* (London: Routledge), 5.

82. A Midland Doctor (1921), *Letters to a Nurse* (London: John Bale, Sons and Danielsson), 52–53.

83. Doctor, 11–15.

84. Eva Gamarnikow (1991), “Nurse or Woman: Gender and Professionalism in Reformed Nursing 1860-1923,” 126–28.

85. Sexual impropriety was discussed by the GNC Disciplinary Committee slightly more often than crimes committed by nurses, most often theft or forgery.

86. Sarah Chaney (2019), “‘Purifying the Profession’: Good Character and the General Nursing Council Disciplinary Committee in the Inter-War Period,” *Women’s History: The Journal of the Women’s History Network* 2, no. 13: 9–13.

87. Disciplinary Committee: Transcripts of Shorthand Notes, 28 November 1927 and 9 January 1934, DT6/45, National Archives, London, UK.

88. Evelyn Clare Pearce (1937), *A General Textbook of Nursing*, 1st edition (London: Faber), 2.

89. Cochrane, *Nursing*, 186.

90. Esther Fisher (1937), *The Nurse’s Textbook: A Manual of Medical and Surgical Nursing*, 1.

91. Douglas Hay Scott (1936), *Modern Professional Nursing, Vol. 2*, 186.

92. E. Maude Smith (1929), *Notes on Practical Nursing*, 16–17.

93. Thomas Dixon (2015), *Weeping Britannia: Portrait of a Nation in Tears*, 229.

94. Pam Smith, 2012, *The Emotional Labour of Nursing Revisited*, 22–23.

95. RCNi (2015), “The 6 Cs of Nursing,” Nursing Standard, 2015, https://rcni.com/revalidation/6cs-of-nursing-32156.

96. As Alison Kitson found when she attempted to formalize concepts of caring in 1993. Alison Kitson (1993), “Formalizing concepts related to nursing and caring” in Alison Kitson, ed. *Nursing: Art and Science* (London: Chapman and Hall)
